# Evaluating the Effect of Cell Culture on Gene Expression in Primary Tissue Samples Using Microfluidic-Based Single Cell Transcriptional Analysis

**DOI:** 10.3390/microarrays4040540

**Published:** 2015-11-04

**Authors:** Michael Januszyk, Robert C. Rennert, Michael Sorkin, Zeshaan N. Maan, Lisa K. Wong, Alexander J. Whittam, Arnetha Whitmore, Dominik Duscher, Geoffrey C. Gurtner

**Affiliations:** Department of Surgery, Division of Plastic and Reconstructive Surgery, 257 Campus Drive West, Hagey Building GK-201, Stanford, CA 94305-5148, USA; E-Mails: januszyk@stanford.edu (M.J.); rrennert@stanford.edu (R.C.R.); msorkin@med.umich.edu (M.S.); zmaan@stanford.edu (Z.N.M.); Lisakanata@gmail.com (L.K.W.); alexander.whittam@gmail.com (A.J.W.); arnethaw@stanford.edu (A.W.); dominikduscher@me.com (D.D.)

**Keywords:** microfluidics, singe-cell, transcriptional analysis, gene expression, bioinformatics, subpopulations, cell culture

## Abstract

Significant transcriptional heterogeneity is an inherent property of complex tissues such as tumors and healing wounds. Traditional methods of high-throughput analysis rely on pooling gene expression data from hundreds of thousands of cells and reporting a population-wide average that is unable to capture differences within distinct cell subsets. Recent advances in microfluidic technology have permitted the development of large-scale single cell analytic methods that overcome this limitation. The increased granularity afforded by such approaches allows us to answer the critical question of whether expansion in cell culture significantly alters the transcriptional characteristics of cells isolated from primary tissue. Here we examine an established population of human adipose-derived stem cells (ASCs) using a novel, microfluidic-based method for high-throughput transcriptional interrogation, coupled with advanced bioinformatic analysis, to evaluate the dynamics of single cell gene expression among primary, passage 0, and passage 1 stem cells. We find significant differences in the transcriptional profiles of cells from each group, as well as a considerable shift in subpopulation dynamics as those subgroups better able to adhere and proliferate under these culture conditions gradually emerge as dominant. Taken together, these findings reinforce the importance of using primary or very early passage cells in future studies.

## 1. Introduction

Functional heterogeneity is an inherent characteristic of complex tissues such as tumors, healing wounds, and various stem cell niches [1,2]. These functional differences are subsumed by variation in both cellular transcriptomics and proteomics [3,4,5]. Although the extent to which single-observation transcriptional or, to a lesser extent, proteomic differences predict physiologic-level functional variations remains controversial [6], significant scientific insights have been obtained though studies evaluating and ultimately validating the functional significance of discrepancies observed at the transcriptional stage [7,8].

Traditional methods of transcriptional analysis, such as DNA microarray, rely on pooling the mRNA from hundreds of thousands of individual cells and reporting a population-wide average. Although this approach has been widely successful at advancing our knowledge of disease physiology over the preceding two decades, these methods lack the granularity to observe differential expression among cellular subsets within the target population [9]. Recent evidence strongly suggests that this limitation severely hampers the utility of such technologies to interrogate the cellular subgroup dynamics inherent to the complex populations described above [10].

Only in the last several years have high-throughput techniques, such as that embodied by the Fluidigm Biomark system, evolved to interrogate tissue with single cell resolution [11]. These approaches make use of microfluidic technology to achieve massively parallel qPCR, permitting the simultaneous interrogation of hundreds of individual cells against large numbers of genes [12]. Such techniques have been successfully employed by our laboratory and others to answer key questions about the fundamental mechanisms underlying stem cell biology, tumor evolution, and the pathobiology of chronic disease [7,13,14,15,16].

One extant question in designing these studies is whether analyzed cells need to be freshly isolated from primary tissue or can be subjected to a priori expansion in cell culture. This is a particularly important issue for scarce populations, such as rare or rarely-resected tumors, for which obtaining sufficient cells is often difficult, whereas heavily passaged commercially available cell lines are readily available. Similarly, rare stem cell populations, such as the putative long-term hematopoietic cell (LT-HSC), which represent only 0.0001% of whole bone marrow, would be more easily studied following culture expansion [17]. Early-gestation embryonic stem cells represent yet another area where the number of cells harvested from a single sample can be as low as four or eight [18]. For these cases, among others, an approach that permits passaging cells in culture before transcriptional interrogation would be considerably attractive. However, the impact of cell culture on single cell transcriptomics remains poorly understood.

In this study, we apply our novel approach to high-throughput, microfluidic-based single cell gene expression analysis to evaluate the effect of cell culture on the transcriptional profiles of stem cells. In particular, we examine one established population of human adipose-derived stem cells, defined as CD45−/CD31−/CD34+, which has been shown to have considerable vasculogenic and angiogenic potential, making it a potentially attractive therapeutic target [14]. We apply partitional clustering, gene enrichment analysis, and network modeling to compare the transcriptional variations observed across culture passaging and evaluate the impact of this *in vitro* expansion.

## 2. Experimental Section

After acquiring informed consent, in accordance with the Stanford University Institutional Review Board guidelines, human abdominoplasty specimens were obtained from a single adult female patient without major medical conditions undergoing an elective abdominoplasty procedure. Adipose-derived stem cells (ASCs) were isolated as described previously by Zuk *et al.* [19]. Briefly, raw samples were manually minced, washed, and treated with 0.075% collagenase type I (Sigma-Aldrich, St. Louis, MO, USA) in Hank’s balanced salt solution (Life Technologies, Grand Island, NY, USA) for 1 h at 37 °C with gentle agitation. The reaction was stopped with the addition of fetal bovine serum (FBS), and after centrifugation, the pelleted stromal vascular fraction was prepared for fluorescence activated cell sorting (FACS) as described below.

Fresh human ASCs were sorted on a FACS Aria II instrument (BD Biosciences, San Jose, CA, USA) with the use of a 100-μm nozzle. Briefly, cells were isolated as described above, and incubated for 20 min in FACS buffer (PBS supplemented with 2% FBS) containing anti-human ef-450-conjugated CD45 (eBioscience, San Diego, CA, USA), allophycocyanin (APC)- or phycoerythrin (PE)-conjugated CD34 (BD Biosciences), and fluorescein isothyocianate (FITC)-conjugated CD31 (BD Biosciences). Using a Becton Dickinson flow cytometric cell sorter, cells were either sorted as single cells into 6 µL of lysis buffer for single cell transcriptional analysis, or as populations for subsequent culture. ASCs were defined with the surface marker profile CD45−/CD31−/CD34+ (to exclude contaminating hematopoietic and endothelial cells found within the stromal vascular fraction (SVF). Cells sorted for culture were plated onto conventional tissue culture plates in the Dulbecco’s modified Eagle’s medium (DMEM; Life Technologies) supplemented with 10% FBS and 1% Penicillin/Streptomycin. Plated cells were cultured under standard conditions (37 °C and 5% CO_2_) and used at passage zero or one.

Single cell reverse transcription and low cycle pre-amplification of individual cells were performed as described previously [20]. Briefly cell suspensions of freshly isolated human SVF were sorted as single cells into each well of a 96-well plate using a Becton Dickinson FACSAria flow cytometer into 6 µL of lysis buffer and SUPERase-In RNAse inhibitor (Applied Biosystems, Waltham, MA, USA). Live/dead gating was performed based on propidium iodide exclusion. Reverse transcription and low cycle pre-amplification was performed following addition of Superscript III reverse transcriptase enzyme (Invitrogen, Waltham, MA, USA), Cells Direct reaction mix (Invitrogen), and target gene-specific TaqMan assay (primer/probe) sets (Applied Biosystems) (Table S1). Resultant single cell cDNA was mixed with sample loading agent (Fluidigm, South San Francisco, CA, USA) and Universal PCR Master Mix (Applied Biosystems) and loaded into 48.48 Dynamic Array chips (Fluidigm) along with TaqMan assays and assay loading agent according to the manufacturer’s instructions. Products were analyzed on the BioMark reader system (Fluidigm) using a hot start protocol to minimize primer-dimer formation, with 40 quantitative PCR cycles performed.

Analysis of single cell data was performed as previously described [8,20]. Briefly, expression data from all chips/passages were normalized relative to the median expression for each gene in the pooled sample and converted to base 2 logarithms. Absolute bounds of ±5 cycle thresholds from the median were set, and non-expressers were assigned to this floor (−5). Clustergrams were then generated using hierarchical clustering in order to facilitate data visualization using MATLAB (R2011b, MathWorks, Natick, MA, USA).

In order to detect subpopulations based on single cell transcriptional data, k-means clustering was employed, assigning each cell to membership in a single cluster as dictated by similarities in expression profiles (minimizing the within-cluster sum of square distances in the 48-dimensional gene hyperspace). Optimally partitioned clusters were then sub-grouped using hierarchical clustering in order to facilitate visualization of data patterning within and across these clusters [20].

Two-sample Kolmogorov-Smirnov (K-S) tests were used to identify genes whose expression patterns differed significantly between population clusters and/or passage groups, using a strict cutoff of *p* < 0.05 following Bonferroni correction for multiple samples. For comparisons among subgroups, the empirical distribution of cells from each cluster was evaluated against that of the remaining cells in the experiment.

Ingenuity Pathway Analysis (IPA, Ingenuity Systems, Redwood City, CA, USA) was used to construct transcriptome networks based on genes that were significantly increased following passage from fresh→P0 and P0→P1, as well as among the 3 individual subpopulations determined above. For this analysis, the 48 genes included in the corresponding single cell analysis (rather than the entire transcriptome) were used as the reference set in order to avoid biasing the associated enrichment calculations in IPA’s internal network generation algorithm.

## 3. Results and Discussion

### 3.1. Cell Culture Alters the Transcriptional Profiles of Human Adipose-Derived Stem Cells

**Figure 1:**
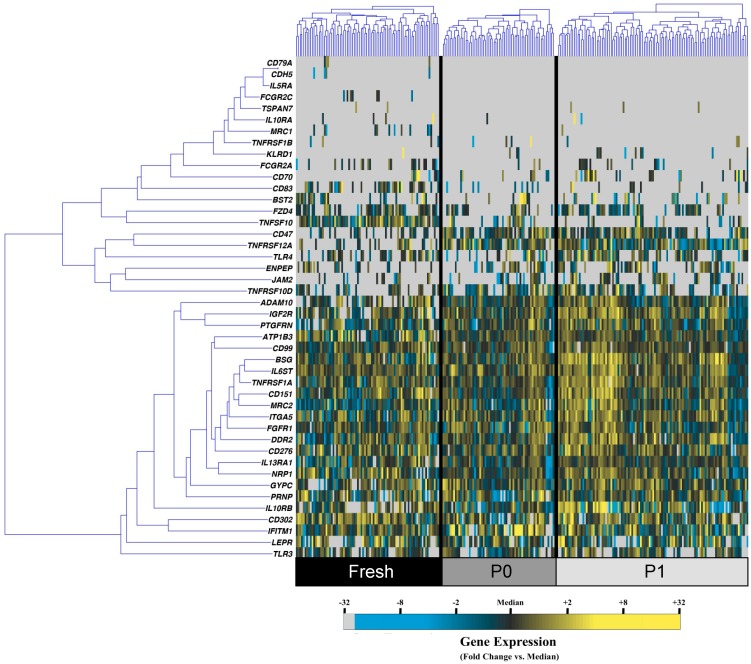
Single cell transcriptional analysis of fresh, passage 0, and passage 1 adipose-derived stem cells. Hierarchical clustering of simultaneous gene expression for single cells from fresh (left; black), passage 0 (middle; dark grey), and passage 1 (right; light grey) human adipose-derived stem cells (ASCs). Gene expression is presented as fold change *vs.* median on a color scale from yellow (high expression, 32-fold above median) to blue (low expression, 32-fold below median). Cell/gene qPCR reactions failing to amplify after 40 cycles are designated as non-expressers and represented in grey.

Utilizing a microfluidic-based single-cell gene expression platform previously described by our laboratory [20], the transcriptional profiles of 60–100 individual cells from fresh, P0, and P1 adipose-derived stem cells (ASCs) were simultaneously evaluated for 48 surface-marker coding gene (Table S1). In this analysis, ASCs isolated from all passages displayed significant heterogeneity at the single-cell level, both within and across groups (Figure 1).

**Figure 2:**
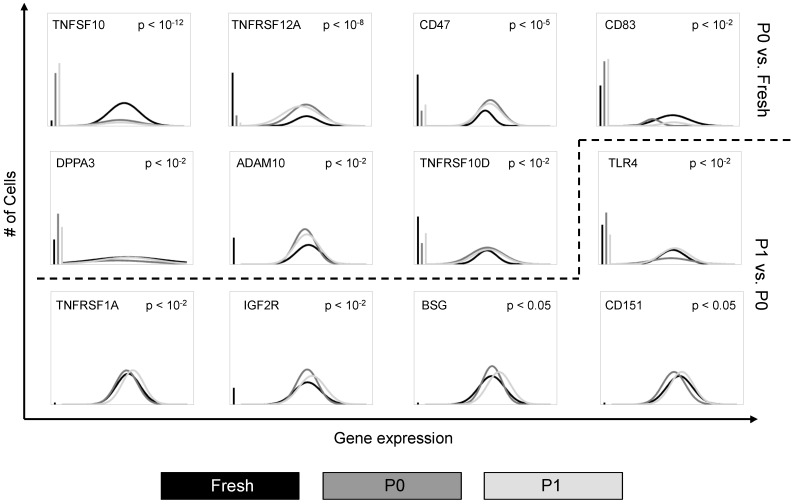
Cell culture alters the transcriptional profile of adipose-derived stem cells. Differentially-expressed genes among primary, passage 0, and passage 1 cells identified using non-parametric two sample Kolmogorov–Smirnov testing. Twelve genes exhibit significantly different (*p* < 0.01 following Bonferroni correction for multiple comparisons) distributions of single cell expression between fresh→P0 or P0→P1 populations, illustrated here using median-centered Gaussian curve fits. The left bar for each panel represents the fraction of qPCR reactions that failed to amplify in each group. The curves above the dashed line represent transcriptional comparisons between primarily isolated cells versus cells at passage 0, whereas the curves below the dashed line represent comparisons between cells at passage 1 versus passage 0.

Differences in the transcriptional profiles of genes related to cell stemness, proliferation, and tumor biology, such as the CD47, CD151, DPPA3, and IGF2R were also observed at the single cell level (Figure 2), suggesting a potential evolution of these properties throughout expansion in cell culture. These differences are embodied in both the fraction of cells expressing these genes, as well as the characteristics of their distributions across cell types.

**Figure 3:**
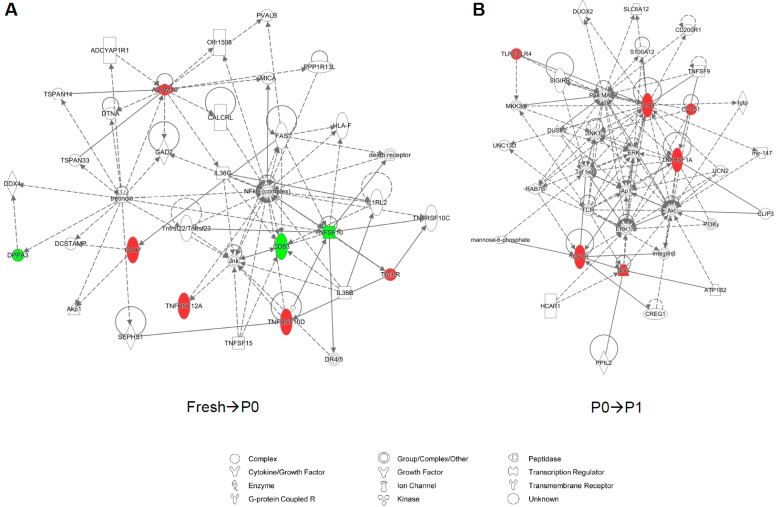
Network analysis of gene expression changes in of fresh, passage 0, and passage 1 adipose-derived stem cells. Top scoring Ingenuity Pathway Analysis (IPA)-constructed transcriptome networks based genes that were significantly up-regulated in fresh→P0 (**A**) and P0→P1 (**B**) human adipose-derived stem cells (ASCs). Direct relationships are indicated by solid lines, and dashed lines represent indirect relationships.

Furthermore, the top molecular networks associated with each set of significantly-altered genes, generated using the Ingenuity Knowledge Base, appear to link key mediators of cell proliferation, embryonic development, and organogenesis (Figure 3).

Collectively, these data support the transcriptional evolution of cultured stem cells toward gene expression profiles characterized by more robust, proliferative, and stem-like properties.

### 3.2. Partitional Cluster Analysis Reveals Subpopulations of Adipose-Derived Stem Cells that Evolve Throughout Cell Culture Passage

**Figure 4:**
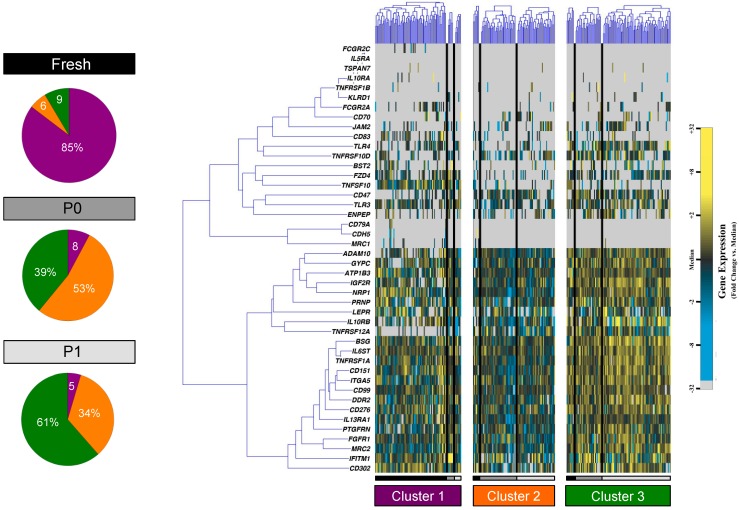
Partitional cluster analysis of single cell adipose-derived stem cell data across cell culture passage. K-means clustering of fresh (black), passage 0 (dark grey), and passage 1 (light grey) human adipose-derived stem cells (ASCs). Gene expression is presented as fold change from median on a color scale from yellow (high expression, 32-fold above median) to blue (low expression, 32-fold below median). Pie graphs represent the fraction of cells comprising each cluster from fresh, passage 0 (P0), and passage 1 (P1) cells, colored in purple, orange, and green in accordance with clusters 1, 2, and 3, respectively.

To further examine this niche, the super-set of transcriptional profiles for primary, passage 0, and passage 1 cells was evaluated using unbiased partitional clustering of gene expression data, as previously described [20]. This analysis identified three distinct, transcriptionally-defined ASC subpopulations or clusters within each phenotypic group (Figure 4). Interestingly, the first subgroup was considerably enriched for primary cells, the second subgroup dominated by a majority of passage 0 cells and a strong minority of passage 1 cells, and the third subgroup comprised primarily of passage 1 cells.

**Figure 5:**
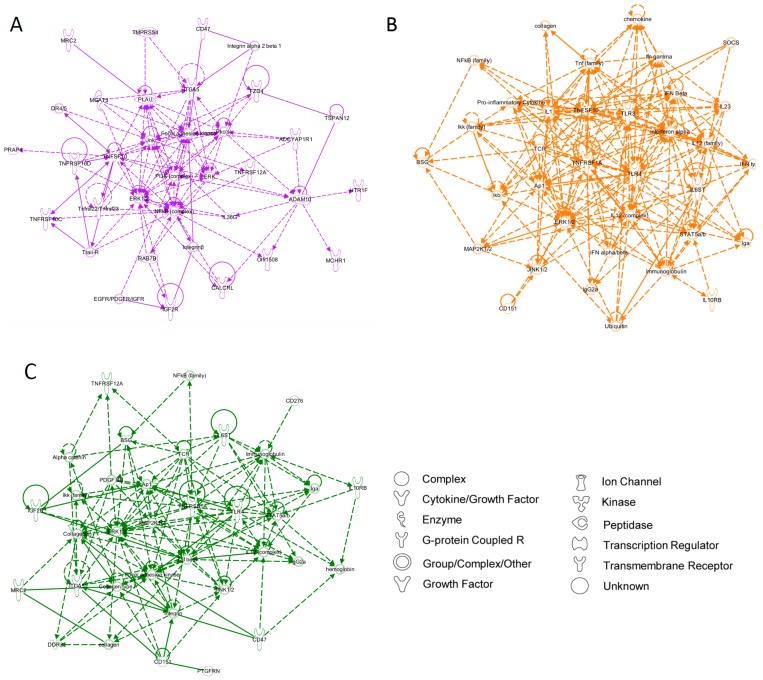
Network analysis of adipose-derived stem cell subpopulations. Top scoring Ingenuity Pathway Analysis (IPA)-constructed transcriptome networks based genes that were significantly up-regulated in cluster 1 (**A**; purple), cluster 2 (**B**; orange), and cluster 3 (**C**; green). Direct relationships are indicated by solid lines, and dashed lines represent indirect relationships.

To predict the functional significance of these subpopulations, we evaluated the top molecular networks associated with genes up-regulated in each cellular subgroup using known transcriptional relationships derived from the Ingenuity Knowledge Base (IPA). The network generated from the first subpopulation (cluster 1), dominated by fresh ASCs, was characterized by non-specific cell-to-cell signaling and interactions (Figure 5A). The second network, derived from cluster 2, was associated with cellular proliferation and inflammatory pathways, which may represent the temporal shift of cells in this subgroup from primary to early passage (Figure 5B), and the third network (corresponding to cluster 3 and dominated by P1/P0 cells) was similarly characterized by cell development, proliferation, and movement (Figure 5C).

To further explore the significance of these signaling pathways, we used IPA to merge these three networks to generate a super-network including all genes associated with either pathway (Figure S1). Interestingly, the central elements linking these networks were extracellular-related kinase (ERK)1/2 and focal adhesion kinase (FAK), both closely implicated in the hyperproliferative response in cutaneous scarring [21], and tumor necrosis factor receptor superfamily member 12A (TNFRSF12A), which is a key regulatory element of cellular invasion in breast cancer [22].

## 4. Conclusions

Functional heterogeneity is a characteristic feature of complex cell populations that is frequently overlooked in studies employing traditional population-averaging measurement techniques. Single cell analytical methods provide the granularity to examine cellular subgroups within these complex samples, but harvesting and isolating sufficient quantities of rare or highly-purified populations is frequently challenging. In this study, we examined the transcriptional consequences of pre-passaging a well-studied human stem cell population prior to gene expression profiling. Our results demonstrate that these cells undergo significant transcriptional changes throughout cell culture as early as passage 0 and passage 1. Further, we observed a considerable shift in subpopulation dynamics, with comparative gene expression profiles suggestive of the gradual emergence of more active and proliferative subgroups. Taken together, these findings reinforce the importance of using primary or very early passage cells in future studies.

## Supplementary Materials

Supplementary materials can be found at http://www.mdpi.com/2076-3905/4/4/540/s1.
