# Supplementary material for: Evaluating the Effect of Cell Culture on Gene Expression in Primary Tissue Samples Using Microfluidic-Based Single Cell Transcriptional Analysis
Source: Microarrays (Basel). 2015 Nov 4;4(4):540–50. doi: 10.3390/microarrays4040540 (PMC4996408; doi:10.3390/microarrays4040540)
Supplement: Supplementary file 1 [file microarrays-04-00540-s001.pdf]

Supplementary Information

Table S1. TaqMan assays used to interrogate gene expression within human adipose-derived stem cells. All assays were obtained from Applied Biosystems (Foster City, CA, USA).

Gene ID	Assay ID	Gene ID	Assay ID
ADAM 10	Hs00153853 m1	IL10RB	Hs00175123 m1
ATP1B3	Hs00740857 mH	IL13RA1	Hs00609817 m1
BSG	Hs00936295 m1	IL5RA	Hs00602482 m1
BST2	Hs00171632 m1	IL6ST	Hs00174360 m1
CD151	Hs00388381 m1	ITGA5	Hs01547673 m1
CD276	Hs00987207 m1	JAM2	Hs01022013 m1
CD302	Hs00994886 m1	KLRD1	Hs00233844 m1
CD47	Hs00179953 m1	LEPR	Hs00174497 m1
CD70	Hs00174297 m1	LIN28	Hs00702808 s1*
CD79A	Hs00233566 m1	MRC1	Hs00267207 m1
CD83	Hs00188486 m1	MRC2	Hs00195862 m1
CD99	Hs00365982 m1	NANOG	Hs02387400 g1*
CDH5	Hs00901463 m1	NRP1	Hs00826128 m1
DDR2	Hs00178815 m1	POU5F1	Hs00999634 gH*
DPPA3	Hs01657068 g1*	PRNP	Hs00175591 m1
ENPEP	Hs00157366 m1	PTGFRN	Hs01385989 m1
FCGR2A	Hs00234969 m1	TLR3	Hs01551078 m1
FCGR2C	Hs00234969 m1	TLR4	Hs00152939 m1
FGFR1	Hs00915142 m1	TNFRSF10D	Hs00388742 m1
FZD4	Hs00201853 m1	TNFRSF12A	Hs00171993 m1
GYPC	Hs00242584 m1	TNFRSF1A	Hs01042313 m1
IFITM1	Hs01652522 g1	TNFRSF1B	Hs00961749 m1
IGF2R	Hs00974474 m1	TNFSF10	Hs00921974 m1
IL10RA	Hs00155485 m1	TSPAN7	Hs00190284 m1

* Assay did not produce exponential amplification, excluded from analysis.

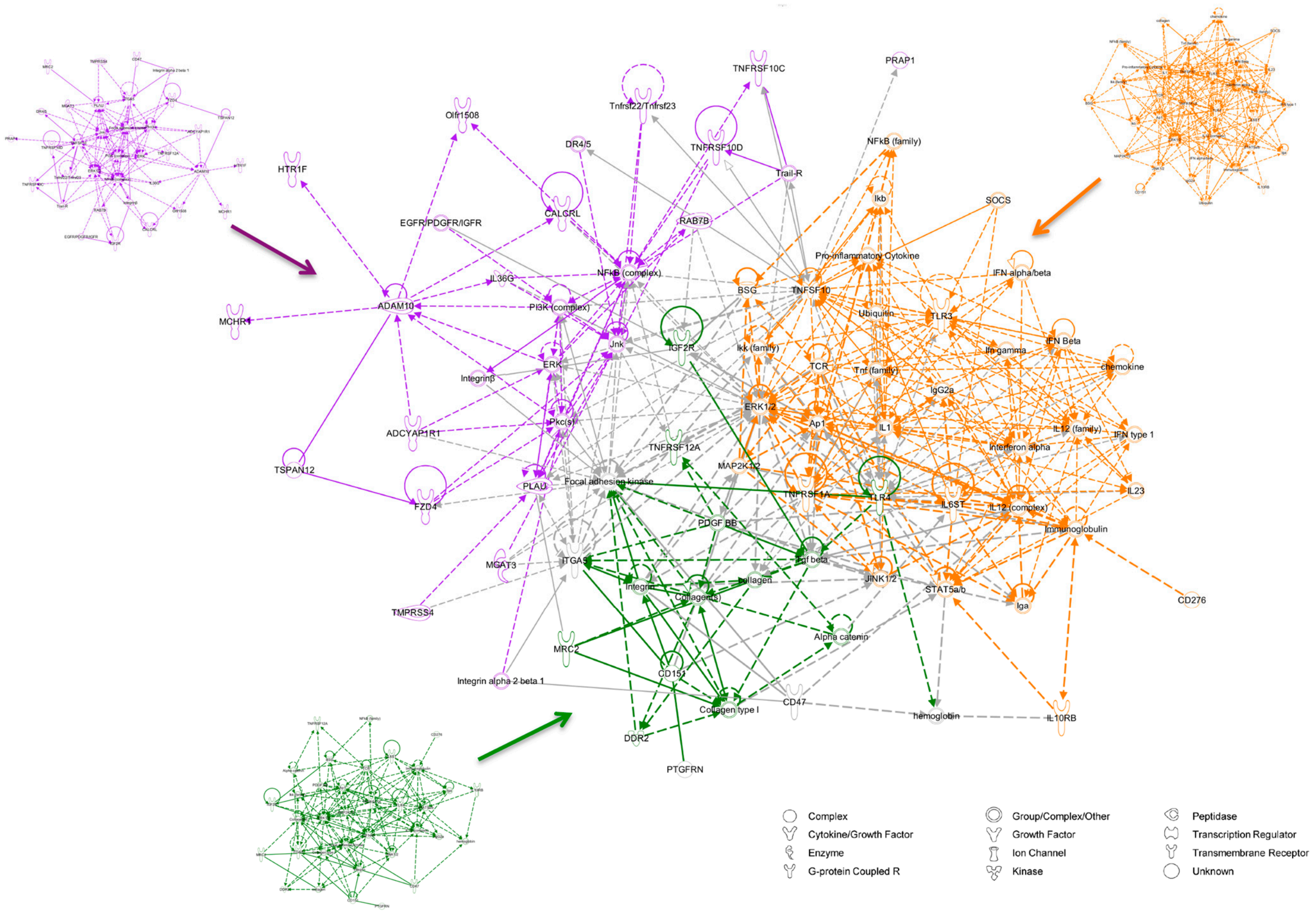

Figure S1. Merged network analysis of fresh, passage 0, and passage 1 adipose-derived stem cells. The top scoring Ingenuity Pathway Analysis (IPA)-constructed transcriptome networks generated from genes that were significantly up-regulated in cluster 1 (purple), cluster 2 (orange), or cluster 3 (green), as enumerated in Figure 5, were merged using IPA's Ingenuity Knowledge Base. Direct relationships are indicated by solid lines, and dashed lines represent indirect relationships.
